# The effect of aerobic exercise on pulse wave velocity in middle-aged and elderly people: A systematic review and meta-analysis of randomized controlled trials

**DOI:** 10.3389/fcvm.2022.960096

**Published:** 2022-08-18

**Authors:** Gen Li, Yuanyuan Lv, Qing Su, Qiuping You, Laikang Yu

**Affiliations:** ^1^Key Laboratory of Physical Fitness and Exercise, Ministry of Education, Beijing Sport University, Beijing, China; ^2^Department of Strength and Conditioning Training, Beijing Sport University, Beijing, China; ^3^China Institute of Sport and Health Science, Beijing Sport University, Beijing, China; ^4^Ersha Sports Training Center of Guangdong Province, Guangzhou, China; ^5^Sports Coaching College, Beijing Sport University, Beijing, China

**Keywords:** aerobic exercise, arterial stiffness, pulse wave velocity, middle-aged people, elderly people

## Abstract

A growing body of research examines the effect of aerobic exercise on pulse wave velocity (PWV) in middle-aged and elderly people, while findings of available studies were conflicting. The aim of this study was to explore the effect of aerobic exercise on PWV in middle-aged and elderly people. Searches were performed in PubMed, Web of Science, and EBSCO databases. Cochrane risk assessment tool was used to evaluate the methodological quality of the included literature. We included studies that satisfied the following criteria: (1) eligible studies should be randomized controlled trials (RCTs); (2) eligible studies should include both an intervention and a control group; (3) eligible studies should use the middle-aged or elderly people as subjects; and (4) eligible studies should use PWV as the outcome measure. From 972 search records initially identified, 11 studies with a total of 12 exercise groups (*n* = 245) and 11 control groups (*n* = 239) were eligible for meta-analysis. There was a significant effect of aerobic exercise on reducing PWV in middle-aged and elderly people [weighted mean difference (WMD), –0.75 (95% CI, –1.21 to –0.28), *p* = 0.002]. Specifically, a higher intensity [vigorous-intensity, –0.74 (–1.34 to –0.14), *p* = 0.02; moderate-intensity, –0.68 (–1.49 to 0.12), *p* = 0.10], a younger age [45 years ≤ age < 60 years, –0.57 (–0.78 to –0.37), *p* < 0.00001; age ≥ 60 years, –0.91 (–2.10 to 0.27), *p* = 0.13], a better health status [healthy, –1.19 (–2.06 to –0.31), *p* = 0.008; diseased, –0.32 (–0.64 to –0.01), *p* = 0.04], and a lower basal body mass index (BMI) [BMI < 25, –1.19 (–2.06 to –0.31), *p* = 0.008; 25 ≤ BMI < 30, –0.52 (–0.92 to –0.12), *p* = 0.01; BMI ≥ 30, –0.09 (–0.93 to 0.76), *p* = 0.84] were associatedwith larger reductions in PWV. Aerobic exercise, especially vigorous-intensity aerobic exercise, contributed to reducing PWV in middle-aged and elderly people. The effect of aerobic exercise on improving PWV was associated with characteristics of the participants. Specifically, a younger age, a better health status, and a lower basal BMI contributed to more significant reductions in PWV.

**Systematic review registration:**

[https://www.crd.york.ac.uk/prospero/display_record.php?ID=CRD42022337103], identifier [CRD42022337103].

## Introduction

With aging, cardiovascular function gradually declines, especially in middle-aged and elderly people, which is mainly manifested as the aging of the cardiovascular and the decline of blood supply. Cardiovascular diseases (CVDs) are the primary chronic disease affecting human health and quality of life. In recent years, with the change of lifestyle, the prevalence and mortality of CVDs have increased year by year. At the same time, the onset age of CVDs also presents a younger trend. CVDs, such as atherosclerosis, congenital and rheumatic heart disease, and hypertension, are the leading cause of disability and death in middle-aged and elderly people, accounting for more than 40% of the deaths in people aged 65 years and above (1).

Arterial stiffness, which is considered to be one of the earliest pathophysiological processes in the progression of atherosclerosis-related metabolic diseases, is a prominent manifestation of vascular aging, and it has become an independent risk factor for atherosclerosis (2), coronary heart disease (3), diabetes (4), stroke (5), and other CVDs (6, 7). Increased arterial stiffness or decreased vascular elasticity can impair arterial function, leading to increased systolic blood pressure, left ventricular hypertrophy, and reduced ventricular diastolic function (8, 9), and thus increases the risk of arteriosclerosis and coronary artery disease (10, 11). Therefore, the prevention and treatment of arterial stiffness is crucial (1, 12).

Previous studies have shown that pulse wave velocity (PWV), especially carotid-femoral pulse wave velocity (cfPWV), has been recommended as the gold standard for assessing arterial stiffness as a non-invasive measurement (8, 11, 12). In addition, PWV is considered to be negatively correlated with vascular health (13). However, these results show more negative outcomes when the blood vessels are stiffer and the lumen is narrowed and thickened, suggesting increased arteriosclerosis, decreased vascular elasticity levels, arteriosclerosis, and an increased risk of coronary artery disease (9). Therefore, reducing PWV is one of the main goals for improving cardiovascular function (14, 15).

Exercise is considered to be an effective measure to prevent CVD and improve its prognosis. In recent years, a large number of studies on exercise and cardiovascular function have shown that appropriate exercise can effectively improve arterial stiffness and reduce the risk of CVDs (10, 12, 16). Older adults who are physically active have less decline in physical function and health and a lower incidence of CVDs than those who are inactive (17, 18). However, there is substantial evidence that high-intensity exercise may lead to CVDs (19). Furthermore, exercise can increase the risk of acute CVDs in sedentary older adults who are not used to sudden high-intensity exercise (19).

At present, there are various intervention methods for middle-aged and elderly people, such as aerobic exercise, resistance exercise, and combined exercise. Totosy de Zepetnek et al. (20) showed that combined exercise had no significant effect on cardiovascular function in middle-aged and elderly people, which is in line with a meta-analysis conducted by Zhang et al. (21). However, aerobic exercise is considered to be an effective method for improving arterial stiffness, whereas the results of resistance exercise are controversial (20). Among different types of intervention methods, aerobic exercise has a higher degree of freedom, is simpler and more convenient, and is more suitable for middle-aged and elderly people. Therefore, aerobic exercise is recommended as the preferred exercise for middle-aged and elderly people (11).

A growing body of research examines the effect of aerobic exercise on PWV in middle-aged and elderly people, while findings of available studies were conflicting. Many studies have confirmed that aerobic exercise can effectively reduce PWV in middle-aged and elderly people (13, 22, 23), while some studies have shown that aerobic exercise has no significant effect on the PWV (16, 19, 24–27). Therefore, we conducted a comprehensive systematic review and meta-analysis of randomized controlled trials (RCTs) to explore whether aerobic exercises have a role in improving PWV in middle-aged and elderly people.

## Methods

This systematic review and meta-analysis was conducted following the guidelines of the Cochrane Selection Manual (28) and the Preferred Reporting Items for Systematic Reviews and Meta-Analysis (PRISMA) (29). The protocol for this systematic review has been registered on PROSPERO (CRD42022337103).

### Search strategy

For this systematic review and meta-analysis, we searched through PubMed, Web of Science, and EBSCO electronic databases from the inception of indexing until October 11, 2021. The initial search contained the following terms: (a) exercise, aerobic exercise, endurance exercise, aerobic training, endurance training, cardio training, physical endurance, physical exertion; (b) pulse wave velocity, PWV, pulse wave analysis, (analyses, pulse wave), (analysis, pulse wave), pulse wave analyses, (wave analyses, pulse), (wave analysis, pulse), pulse wave velocity, pulse wave velocities, (velocities, pulse wave), (velocity, pulse wave), (wave velocities, pulse), (wave velocity, pulse), pulse transit time, pulse transit times, (time, pulse transit), (times, pulse transit), (transit time, pulse), (transit times, pulse), pulse wave transit time; (c) elderly, aged, geriatrics, eldest, old, older, middle-aged, middle aged, middle age, elderly, aged, geriatrics, eldest, old, older, middle-aged, middle aged, middle age. We also hand-searched reference lists of all identified studies. We excluded studies based on the review of the title, abstract, and full text. Two authors (GL and YL) conducted the process independently using a standardized form. In case of any discrepancies between the two authors, a third author (LY) was involved in the discussion until a consensus was made.

### Eligibility criteria

We included studies that satisfied the following criteria: (1) eligible studies should be RCTs; (2) eligible studies should include both an intervention and control group with the only difference between them being the addition of aerobic exercise in the intervention group; (3) eligible studies should use the middle-aged or elderly people as subjects; and (4) eligible studies should use cfPWV as the outcome measure. Non-English language publications, animal model publications, reviews, and conference articles were excluded from the analysis.

### Data extraction

Two authors (GL and YL) of this study performed the data extraction independently using the same and standardized form created in Microsoft Excel. If there were any discrepancies between the authors in the extracted data, the accuracy of the information was rechecked in the studies. The extracted variables mainly included: (a) characteristics of included studies (first author’s last name, year of study publication); (b) characteristics of aerobic exercise (type of exercise, intensity, duration of intervention, session duration, frequency); (c) participant’s characteristics [n, age, gender, basal body mass index (BMI), basal systolic blood pressure (SBP), basal diastolic blood pressure (DBP), health status]; and (d) treatment effects [mean and standard deviation (SD) values reflecting the change in PWV from baseline to post-intervention in the aerobic exercise and control groups].

### Methodological quality assessment

We assessed the methodological quality of the included studies using the Cochrane risk of bias criteria, which included seven items, namely, randomization sequence generation (selection bias), allocation concealment (selection bias), blinding of participants and personnel (performance bias), blinding of outcome assessment (detection bias), incomplete outcome data (attrition bias), selective reporting (reporting bias), and other bias. Each item was judged as “low risk,” “unclear risk,” or “high risk” based on responses to the signaling questions, to make an overall bias judgment for the specific study outcome being assessed (28). Two reviewers (GL and YL) performed the methodological quality assessment independently. Disagreements in the assessments between the reviewers were resolved through discussion and consensus with a third author (LY).

### Statistical analysis

The mean and SD values reflecting the change in PWV from baseline to post-intervention were extracted from each study for pooling effects. SD was calculated using a previously described formula for studies reporting standard error (SE) or 95% confidence intervals (CIs) (30). When the data could not be extracted or there was a dispute, two authors negotiated or contacted the author of the article to resolve it. Otherwise, the platform was used to extract the information (31).

The majority of included studies provided mean and SD values before and after the intervention. We calculated the changes in the mean and SD values for PWV. When analyzing whether aerobic exercise could improve the PWV of middle-aged and elderly people, the Chi-square (χ^2^) test was used. The negative value of *I*^2^ was defined as zero, so the value of *I*^2^ was between 0 and 100%. As mentioned by Chiarito et al. (32), an *I*^2^ value of 0% indicates no observed heterogeneity, and larger values indicate increasing heterogeneity. The *I*^2^ values of < 25%, 25–75%, and > 75% were considered to represent low, moderate, and high levels of heterogeneity, respectively (33). If there was a high level of heterogeneity (*I*^2^ ≥ 50%), we used subgroup analyses to interpret the results (29). When *I*^2^ is < 50%, data were pooled using fixed effects models to obtain the weighted mean difference (WMD) and 95% CIs; when *I*^2^ is ≥ 50%, data were pooled using random effects models to obtain the WMD and 95% CIs. In the subgroup analyses, we tried to use intensities of aerobic exercise (moderate-intensity and vigorous-intensity), age of participants (45 years ≤ age < 60 years and age ≥ 60 years), basal BMI (BMI < 25, 25 ≤ BMI < 30, and BMI ≥ 30), and health status (healthy and diseased) to explore the impact on PWV. The analysis result, funnel plot, and forest chart were generated using the software RevMan.5. In terms of overall impact, *p* < 0.05 was considered statistically significant.

## Results

### Study selection

**FIGURE 1:**
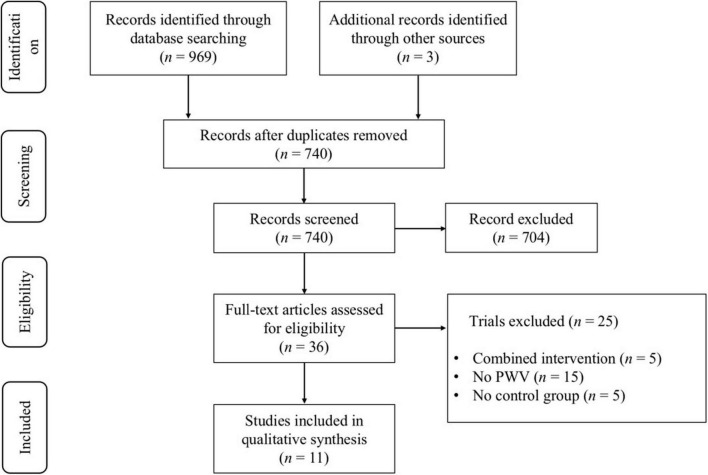
PRISMA flowchart of study selection.

As shown in Figure 1, a total of 969 search records were preliminarily retrieved, and three records were identified through other sources. After excluding the duplicates, 740 studies were remaining, and 704 studies were not eligible for inclusion through the title and abstract screening. Twenty-five studies were excluded by reading the full text of 36 studies: (1) the experimental group combined with other treatments (*n* = 5); (2) outcomes were not relevant (*n* = 15); and (3) no control group (*n* = 5). Finally, 11 studies (13, 19, 22, 23, 25, 27, 34–38) examining the effect of aerobic exercise on PWV in middle-aged and elderly people were considered eligible for systematic review and meta-analysis.

### Characteristics of the included studies

The main characteristics of the participants and exercise interventions are shown in Supplementary Table 1. The included studies involved 245 participants in the 12 exercise groups and 239 participants in the 11 control groups. One study (22) involved only women, and the other 10 studies (13, 19, 23, 25, 27, 34–38) involved both men and women. Three studies (22, 23, 27) involved healthy participants, and eight studies (13, 19, 25, 34–38) involved diseased participants. The average age of the participants was ranging from 45 to 74.3 years. Among them, seven studies (13, 22, 25, 34, 36–38) involved participants with an average age of < 60 years, and four studies (19, 23, 27, 35) involved participants with an average age of ≥ 60 years. There studies (22, 23, 27) involved participants with basal BMI < 25, five studies (19, 25, 34, 37, 38) involved participants with 25 ≤ BMI < 30, and two studies (13, 35) involved participants with BMI > 30.

According to the position statement of physical activity and training intensity (39), we adjusted the intensity classification of aerobic exercise according to the included research situation: 20% < maximal oxygen uptake (VO_2max_) ≤ 40%, 40% < maximum heart rate (HR_max_) ≤ 55%, or 20% < heart rate reserve (HRR) ≤ 40% were determined as light-intensity; 40% < VO_2max_ ≤ 60%, 55% < HR_max_ ≤ 70%, or 40% < HRR ≤ 60% were determined as moderate-intensity; 60% < VO_2max_ ≤ 85%, 70% < HR_max_ ≤ 90%, or 60% < HRR ≤ 85% were determined as vigorous-intensity. Among the included studies, the intensity of two studies (19, 36) could not be defined, two studies (13, 34) were defined as moderate-intensity, and seven studies (22, 23, 25, 27, 35, 37, 38) were defined as vigorous-intensity.

### Risk of bias

**FIGURE 2:**
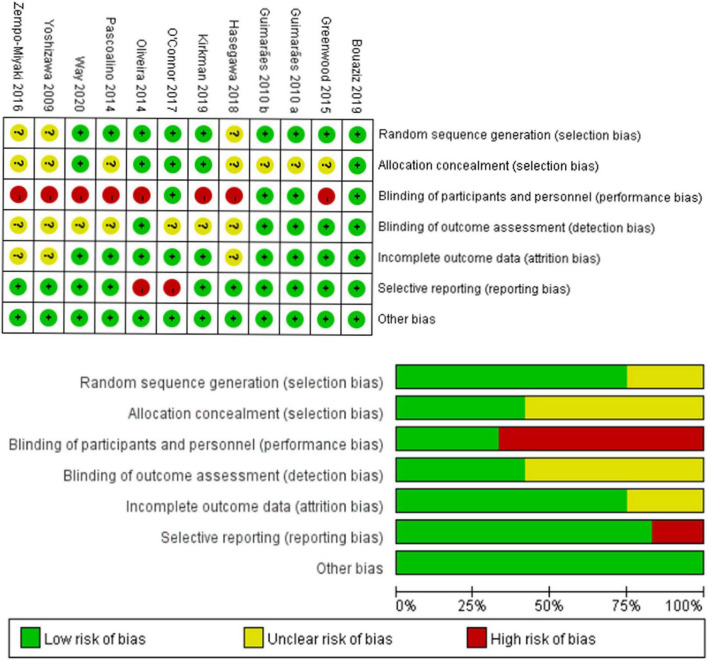
Results of Cochrane risk of bias tool.

**FIGURE 3:**
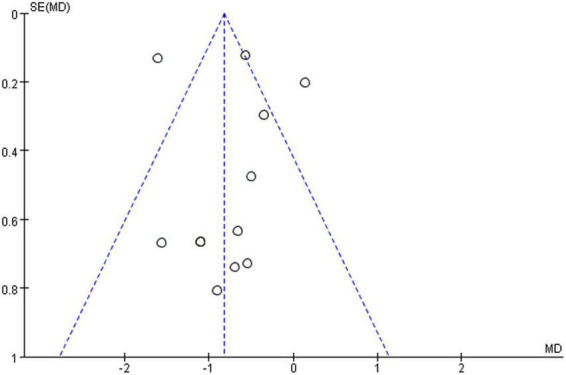
Funnel plot.

Cochrane risk assessment tool was used to evaluate the methodological quality of the included literature, mainly from six aspects, namely, selection bias, performance bias, detection bias, attrition bias, reporting bias, and other bias. The quality score was determined according to three levels (low risk, high risk, and unclear). The quality of the included literature was divided into three levels from high to low, namely, high quality, medium quality, and low quality (see Figure 2). Publication bias was assessed visually by inspecting the funnel plot (see Figure 3).

### Meta-analysis results

#### Effects of aerobic exercise on pulse wave velocity

**FIGURE 4:**
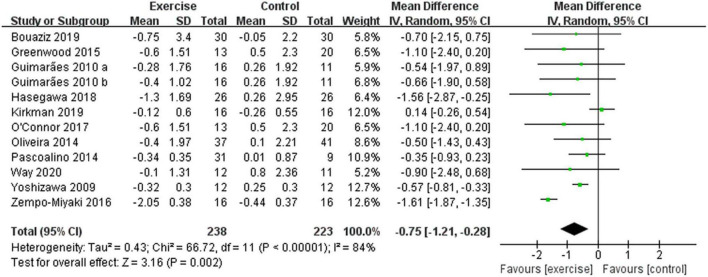
Meta-analysis results of the effect of aerobic exercise on the PWV of middle-aged and elderly people. The pooled estimates were obtained from random effects analysis. Diamonds indicate the effect size of each study summarized as WMD. The size of the shaded squares is proportional to the percentage weight of each study. Horizontal lines represent the 95% CI and the vertical line represents the overall effect.

After analyzing the data of all included studies, we found that compared with the control group, aerobic exercise had a significant effect on reducing PWV in middle-aged and elderly people [WMD, –0.75 (95% CI, –1.21 to –0.28), *p* = 0.002], while there was a high heterogeneity (*I*^2^ = 84%) (Figure 4). Therefore, we used subgroup analyses to interpret the results.

#### Subgroup analysis

**FIGURE 5:**
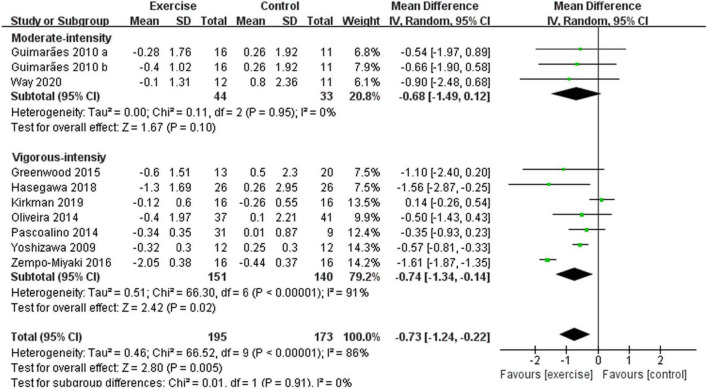
Meta-analysis results of the effect of different intensities of aerobic exercise on the PWV of middle-aged and elderly people. The pooled estimates were obtained from random effects analysis. Diamonds indicate the size of the effect of each study summarized as WMD. The size of the shaded square is proportional to the percentage weight of each study. Horizontal lines represent the 95% CI and the vertical dashed line represents the overall effect.

Different results were shown when considering exercise intensity (see Figure 5). Specifically, compared with the control group, vigorous-intensity exercise significantly reduced PWV [WMD, –0.74 (95% CI, –1.34 to –0.14), *p* = 0.02, *I*^2^ = 91%], while moderate-intensity exercise had no significant effect on PWV in middle-aged and elderly people [WMD, –0.68 (95% CI, –1.49 to 0.12), *p* = 0.10, *I*^2^ = 0%]. Subgroup analysis indicated that a higher intensity was associated with larger reductions in PWV.

**FIGURE 6:**
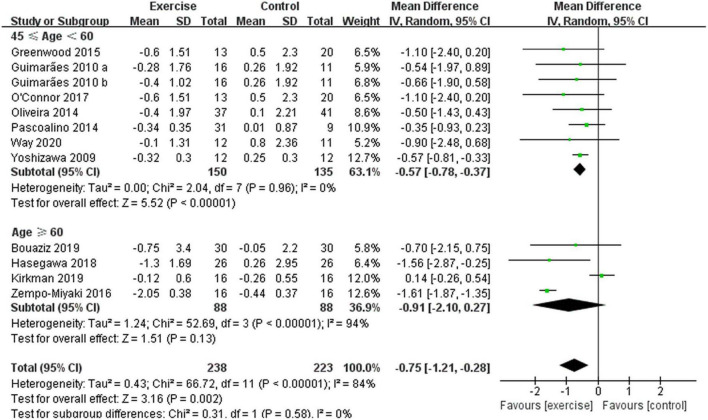
Meta-analysis results of the effect of aerobic exercise on the PWV of middle-aged people or elderly people. The pooled estimates were obtained from random effects analysis. Diamonds indicate the size of the effect of each study summarized as WMD. The size of the shaded square is proportional to the percentage weight of each study. Horizontal line represents the 95% CI and the vertical dashed line represents the overall effect.

**FIGURE 7:**
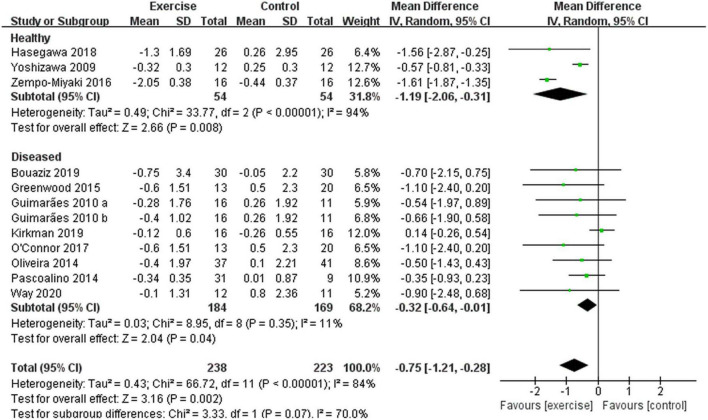
Meta-analysis results of the effect of aerobic exercise on the PWV of healthy or diseased middle-aged and elderly people. The pooled estimates were obtained from random effects analysis. Diamonds indicate the size of the effect of each study summarized as WMD. The size of the shaded square is proportional to the percentage weight of each study. Horizontal line represents the 95% CI and the vertical dashed line represents the overall effect.

**FIGURE 8:**
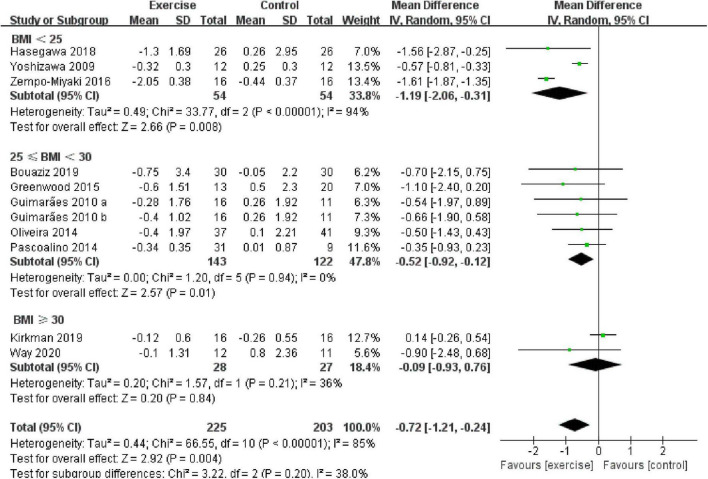
Meta-analysis results of the effect of aerobic exercise on the PWV of normal weight, overweight, or obese middle-aged and elderly people. The pooled estimates were obtained from random effects analysis. Diamond indicated the size of the effect of each study summarized as WMD. The size of the shaded square is proportional to the percentage weight of each study. Horizontal line represents the 95% CI and the vertical dashed line represents the overall effect.

In addition, different results were shown when considering participants’ characteristics. The subgroup analysis indicated that a younger age [45 years ≤ age < 60 years, –0.57 (95% CI, –0.78 to –0.37), *p* < 0.00001, *I*^2^ = 0%; age ≥ 60 years, –0.91 (95% CI, –2.10 to 0.27), *p* = 0.13, *I*^2^ = 94%. see Figure 6], a better health status [healthy, –1.19 (95% CI, –2.06 to –0.31), *p* = 0.008, *I*^2^ = 94%; diseased, –0.32 (95% CI, –0.64 to –0.01), *p* = 0.04, *I*^2^ = 11%. see Figure 7], and a lower basal BMI [BMI < 25, –1.19 (95% CI, –2.06 to –0.31), *p* = 0.008, *I*^2^ = 94%; 25 ≤ BMI < 30, –0.52 (95% CI, –0.92 to –0.12), *p* = 0.01, *I*^2^ = 0%; BMI ≥ 30, –0.09 (95% CI, –0.93 to 0.76), *p* = 0.84, *I*^2^ = 36%. see Figure 8] were associated with larger reductions in PWV.

## Discussion

### Effects of aerobic exercise on pulse wave velocity

This systematic review and meta-analysis indicated that aerobic exercise had the potential to reduce arterial stiffness in middle-aged and elderly people, as manifested by a reduction in PWV. Previous studies were consistent with our results, for example, 10 weeks of shallow water aerobic exercise significantly reduced PWV in elderly people (40), 20 weeks of walking training significantly reduced PWV in elderly people (41), and 21 weeks of aerobic exercise effectively reduced PWV in kidney transplant recipients (25). However, the mechanism by which aerobic exercise improves arterial stiffness has not been fully revealed.

Many studies have shown that the lack of exercise may lead to fat accumulation and disorders of glycolipid metabolism (42–44). Adipose tissue produces the pro-inflammatory cytokine interleukin-6 (IL-6), tumor necrosis factor-α (TNF-α), plasminogen activator inhibitor, and adiponectin. Therefore, the accumulation of fat may be related to the increased inflammatory response during atherosclerosis, which is consistent with the study of Jennersjö et al. (45), and that the inflammatory response can accelerate the process of arteriosclerosis (46, 47). Previous studies have shown that regular aerobic exercise can reduce fat content and increase adiponectin secretion, which in turn reduces arterial stiffness (27, 30, 48). In addition, a previous study also showed that exercise can reduce risk factors for arterial stiffness associated with metabolic disorders (20). Metabolic syndrome, which can lead to impaired glucose tolerance, hypertension, dyslipidemia, and abdominal obesity, has been reported to be associated with changes in the arterial system, mainly manifested by changes in vascular structure and function, including increased arterial wall thickness and increased vascular wall stiffness (46, 47, 49). Above all, metabolic disorders can accelerate arterial aging through elastin fiber fragmentation, increased pressure on the collagen fibers of the arterial wall (50), and vascular damage, ultimately leading to increased arterial stiffness (51, 52). Aerobic exercise has been shown to have a dampening effect on metabolic disorders (53). There is increasing evidence that aerobic exercise promotes the clearance of triacylglycerol and low-density lipoprotein cholesterol (54–56), thereby preventing oxidative stress, which in turn reduces arterial stiffness.

In addition, Beck et al. (57) found that an increase in PWV may be associated with a decrease in endothelial function. The increase in PWV is manifested by the decrease in the bioavailability of endothelial-derived nitric oxide (NO) (58). NO is produced by L-arginine through endothelial nitric oxide synthase (eNOS) in the vascular endothelium, which has the effect of dilating blood vessels. Maeda et al. suggested that exercise increases the bioavailability of NO and reduces PWV (58), which was in line with the report by Taddei et al. showing that regular exercise restores the utilization of NO after oxidative stress and prevents age-induced endothelial dysfunction. A previous study showed that NO increased and PWV decreased after 24 weeks of aerobic exercise, and a significant negative correlation between NO and PWV was observed (59). Taken together, aerobic exercise in middle-aged and elderly people can increase the synthesis and bioavailability of NO, reduce circulating endothelin-1 (ET-1), increase flow-mediated dilation (FMD) of the brachial artery, improve the endothelial function, and ultimately reduce PWV (60).

In conclusion, aerobic exercise in middle-aged and elderly people can reduce fat accumulation and improve glycolipid metabolism and endothelial function, thereby reducing arterial stiffness and playing an important role in preventing arteriosclerosis.

### Subgroup analysis

According to the studies we included, aerobic exercise significantly reduced PWV in middle-aged and elderly people, but the heterogeneity between groups was high. Therefore, we used subgroup analysis to interpret the results. In the subgroup analyses, we sought to determine the effects of exercise intensity and characteristics of the participants.

Previous studies have demonstrated that aerobic exercise has a positive effect on arterial stiffness (61–63). However, some studies have found that PWV does not always decrease with increasing aerobic exercise intensity, for example, Zempo-Miyaki et al. (23) showed that 8 weeks of aerobic exercise (60–70% peak oxygen uptake) significantly reduced PWV in middle-aged and elderly people, while Oudegeest-Sander et al. (64) reported that 12 months of cycling exercise (70–85% of individual HRR) had no effect on PWV in elderly people, suggesting that exercise intensity was a key factor affecting the impact of the intervention. Therefore, we divided the study into a moderate-intensity group and a vigorous-intensity group. Compared with the control group, vigorous-intensity exercise significantly reduced PWV, while moderate-intensity exercise had no significant effect on PWV. Previous studies have demonstrated that vigorous-intensity exercise may generate greater shear stress in endothelial cells, thereby improving endothelial function, reducing oxidative stress, and improving vascular function (25, 50, 65, 66), which is consistent with our study showing that vigorous-intensity exercise can significantly reduce the arterial stiffness in middle-aged and elderly people. However, recent studies have shown that exercise intensity definition based on percentages of peak HR and VO_2_peak may misclassify the effective exercise intensity, and the discrepancy between the individually determined and the recommended exercise intensity is particularly relevant in cardiac patients. And a ventilatory threshold-based rather than a range-based approach is advisable to define an appropriate level of exercise intensity (67, 68). In addition, according to the European Association of Preventive Cardiology (EAPC) position statement on the assessment and prescription of aerobic exercise intensity in cardiovascular rehabilitation, the assessment of ventilatory threshold 1 (VT1) and VT2 during cardiopulmonary exercise test (CPET) should be used for the determination of the aerobic exercise intensity in the majority of CVD patients (69). Therefore, since studies included in this systematic review and meta-analysis used HR and VO_2_max to define the exercise intensity, those focused on CVD populations need to be cautious when referring to our finding.

A previous study showed that blood pressure increases more rapidly in adults after the age of 60 years, which is the critical point for a higher incidence of CVDs (70). Therefore, the included studies in this meta-analysis were divided into two subgroups by the age of participants, namely, middle-aged group and elderly group. Compared with the control group, aerobic exercise significantly reduced PWV in middle-aged people, while aerobic exercise had no significant effect on PWV in elderly people, indicating that aerobic exercise had a better effect on PWV in middle-aged people than in elderly people. Aging is an inevitable part of life, and aerobic exercise has a limited effect on improving vascular endothelial function. For example, Ha et al. (11) found that 12 weeks of aerobic exercise did not reduce PWV in women aged 70–80 years, which may be related to sex hormone levels of middle-aged and elderly people. Cross-sectional studies have also reported an age-related decline in sex hormones, with a sharp decline in sex hormones after the age of 65 years (71, 72). Additionally, a previous study reported that sex hormone levels may influence the effect of aerobic exercise on improving cardiovascular function (73). In addition, one study found that the benefit of aerobic exercise on vascular function was diminished in estrogen-deficient postmenopausal women, but in this group, estrogen treatment appeared to restore improvements in endothelial function (73). Furthermore, studies have shown that aortic PWV increases by approximately 0.10 m/s per year with age, with a weak annual increase in PWV (less than 0.10 m/s) in subjects before the age of 45 years, indicating a low rate of arteriosclerosis in young adults (74, 75). Therefore, the insignificant decline in PWV in the elderly may also be due to the insufficient effect of exercise against vascular aging.

In the abovementioned results, we suspected that the improvement of arterial stiffness by exercise was influenced by a variety of CVDs, so this report divided the included studies into two subgroups, namely, the healthy group and the diseased group. Compared with the control group, aerobic exercise significantly reduced PWV in both healthy and diseased individuals, while a better health status was associated with larger reductions, which was consistent with a previous study, showing that aerobic exercise had no long-term effect on arterial stiffness in older adults with cardiometabolic risk factors (76). Therefore, the lesser the number of risk factors for CVDs or the types of CVDs, the better the effect of aerobic exercise.

Obesity is an independent predictor of CVDs, and weight loss has been shown to improve many obesity-related risk factors; therefore, we divided the included studies into normal weight, overweight, and obese groups based on the basal BMI of the participants. Our results showed that compared with the control group, aerobic exercise significantly reduced PWV in normal weight and overweight people, whereas aerobic exercise had no significant effect on PWV in obese people. In addition, a lower BMI was associated with larger reductions in PWV, indicating that aerobic exercise had a better effect on PWV in lower BMI people than in higher BMI people, which was consistent with a previous study, showing that a mean weight loss of 8% resulted in a statistically and clinically significant PWV reduction of 0.6 m/s, suggesting that a lower BMI was associated with lower rates of arteriosclerosis (77). Therefore, arteriosclerosis in people with a higher BMI may be a major determinant of morbidity and mortality in this population.

### Limitations of the review

Some potential limitations of this meta-analysis should be acknowledged. First, the included studies were all RCTs of aerobic exercise intervention, which could not be completely blinded. Therefore, in the quality evaluation process, subjective factors will cause a certain degree of deviation. Second, studies included in this systematic review and meta-analysis used HR and VO_2_max to define the exercise intensity, and studies focused on CVDs populations need to be cautious when referring to our finding. Finally, although the included studies did not specify any adverse events associated with the aerobic exercise intervention, it is unclear whether the researchers attempted to comprehensively document all possible adverse events. Therefore, future studies with more detailed data describing possible injury, pain, and/or any other potential adverse effects are encouraged, as this will expand our knowledge of the safety of aerobic exercise in middle-aged and elderly people.

## Conclusion

Our analysis indicated that aerobic exercise, especially vigorous-intensity aerobic exercise, contributed to reducing PWV in middle-aged people. The effect of aerobic exercise on improving PWV was associated with characteristics of the participant. Specifically, a younger age, a better health status, and a lower basal BMI contributed to more significant reductions in PWV.

## Data availability statement

The original contributions presented in this study are included in the article/Supplementary material, further inquiries can be directed to the corresponding author.

## Author contributions

GL wrote the manuscript. LY and YL contributed to the conception. GL and YL did the literature search. GL, YL, and LY extracted the data. QS and QY contributed to the acquisition. All authors have read and approved the final manuscript.
