# Supplementary material for: The effect of aerobic exercise on pulse wave velocity in middle-aged and elderly people: A systematic review and meta-analysis of randomized controlled trials
Source: Front Cardiovasc Med. 2022 Aug 18;9:960096. doi: 10.3389/fcvm.2022.960096 (PMC9433655; doi:10.3389/fcvm.2022.960096)
Supplement: Supplementary file 1 [file Table_1.DOCX]

Supplementary Material

# Supplementary Table 1. Basic characteristics of studies included in the meta-analysis

| Studies | Health status | Male% | Control | |  | Exercise | | Basal BMI (kg/m^2^) | Basal SBP (mmHg) | Basal DBP (mmHg) | Type of exercise | Session duration (min) | Frequency (times/week) | Intensity | Duration of intervention (week) | Outcome | Effect |
| --- | --- | --- | --- | --- | --- | --- | --- | --- | --- | --- | --- | --- | --- | --- | --- | --- | --- |
|  |  |  | *n* | Age |  | *n* | Age |  |  |  |  |  |  |  |  |  |  |
| Bouaziz et al., 2019 | Random population | 73 | 30 | 74.3 ± 3.4 |  | 30 | 72.9 ± 2.5 | 28.7 ± 5.6 | 133.7 ± 9.8 | 80.2 ± 7.0 | Cycling | 30 | 2 | VT_1_ | 9.5 | cfPWV | Nonsignificant change |
| Greenwood et al., 2015 | Kidney transplant | 61 | 20 | 49.56 ± 10.6 |  | 13 | 53.96 ± 10.7 | 26.6 ± 4.7 | 136.8 ± 14.4 | 77.6 ± 13.7 | Cycling/ treadmill | 30 | 3 | 80%HRR | 12 | cfPWV | Decrease |
| Guimarães et al., 2010 a | Hypertension | 33 | 11 | 47 ± 6 |  | 16 | 50 ± 8 | 28 ± 4 | 124 ± 8 | 80 ± 9 | Treadmill | 40 | 3 | 60%HRR | 16 | cfPWV | Nonsignificant change |
| Guimarães et al., 2010 b | Hypertension | 75 | 11 | 47 ± 6 |  | 16 | 45 ± 9 | 29 ± 5 | 125 ± 8 | 80 ± 5 | Treadmill | 40 | 3 | 60%HRR | 16 | cfPWV | Decrease |
| Hasegawa et al., 2018 | Healthy | 50 | 26 | 66.1 ± 8.6 |  | 26 | 68.2 ± 7.1 | 23.5 ± 3.5 | 129.2 ± 18.7 | 76.2 ± 11.6 | Cycling | 55 | 3 | 60-70% VO_2_peak | 8 | cfPWV | Decrease |
| Kirkman et al., 2019 | Chronic kidney disease | 69 | 16 | 62 ± 9 |  | 16 | 55 ± 13 | 30 ± 2 | 138 ± 19 | 82 ± 15 | Cycling/ walking/ jogging | 45 | 3 | 60-85% HRR | 12 | cfPWV | Nonsignificant change |
| O’Connor et al., 2017 | Kidney transplantation | 63 | 46 | 49.5 ± 10.6 |  | 13 | 53.9 ± 10.7 | - | 136.8 ± 14.4 | 77.6 ± 13.7 | - | 30 | 1 | - | 12 | cfPWV | Decrease |
| Oliveira et al., 2014 | Myocardial infarction | 84 | 42 | 58.5 ± 10.7 |  | 44 | 55.0 ± 10.7 | 26.1 (24.5-29.1) | 121.0 (108.5-137.7) | 71.3 ± 8.6 | Cycling/ treadmill | 50 | 3 | 70-85% HR_max_ | 8 | cfPWV | Decrease |
| Way et al., 2020 | T2DM | 57 | 11 | 51.9 ± 1.4 |  | 12 | 54.8 ± 2.4 | 34.3 ± 3.7 | 125 ± 11 | 79 ± 5 | Cycling | 55 | 3 | 60% VO_2_peak | 12 | cfPWV | Decrease |
| Pascoalino et al., 2014 | Heart transplantation | 70 | 9 | 45 ± 6 |  | 31 | 45 ± 3 | 26.7 ± 0.9 | 120 ± 3 | 81 ± 3 | Walking/ jogging | 40 | 3 | 80% VO_2max_ | 12 | cfPWV | Nonsignificant change |
| Yoshizawa et al., 2009 | Healthy | 0 | 12 | 49 ± 3 |  | 12 | 47 ± 2 | 24.6 ± 1.1 | 120 ± 3 | 89 ± 3 | Cycling | 30 | 2 | 60-70% VO_2max_ | 12 | cfPWV | Decrease |
| Zempo-Miyaki et al., 2016 | Healthy | 34 | 16 | 67.4 ± 1.5 |  | 16 | 65.0 ± 2.1 | 24.4 ± 1.1 | 128 ± 5 | 77 ± 3 | Cycling | 55 | 3 | 60-70% VO_2_peak | 8 | cfPWV | Decrease |

T2DM, type 2 diabetes mellitus; VT_1_, first ventilatory threshold; VO_2max_, maximal oxygen consumption; HR_max_, maximal heart rate; HRR, heart rate reserve; VO_2_peak, peak uptake oxygen; cfPWV, carotid-femoral pulse wave velocity.
